# Sulfur, fresh cassava root and urea independently enhanced gas production, ruminal characteristics and *in vitro* degradability

**DOI:** 10.1186/s12917-021-02999-3

**Published:** 2021-09-09

**Authors:** Phussorn Sumadong, Anusorn Cherdthong, Sarong So, Metha Wanapat

**Affiliations:** Tropical Feed Resources Research and Development Center (TROFREC), Department of Animal Science, Faculty of Agriculture, Khon Kaen University, 40002 Khon Kaen, Thailand

**Keywords:** Sulfur, Hydrogen cyanide, Gas production, Ammonia nitrogen, Propionic acid, Degradability

## Abstract

**Background:**

Total fresh cassava root (FCR) production was 275 million tonnes in 2018 which equals 61.1 % of the total production, and Thailand produced 10.7 % FCR of the total production. FCR is one of the main energy source for ruminant. The limitation of FCR utilization is due to the presence of hydrogen cyanide (HCN). The study aimed to evaluate the effect of sulfur, urea and FCR at various levels on *in vitro* gas production, ruminal fermentation and *in vitro* degradability. The study hypothesized that: (1) sulfur, urea and FCR have no interaction effect and (2) effect of FCR and urea is related to sulfur addition.

**Results:**

The study aimed to elucidate the optimum level of elemental sulfur, fresh cassava root (FCR) and urea and their effect on *in vitro* gas production, ruminal fermentation, thiocyanate concentration, and *in vitro* degradability. A 3 × 2 × 4 in a completely randomized design were conducted. Factor A was level of sulfur at 0 %, 1 and 2 % of concentrate dry matter (DM), factor B was level of urea at 2 and 4 % of concentrate DM, and factor C was level of the FCR at 0, 200, 300 and 400 mg DM of the total substrate. The study found that elemental sulfur, urea and FCR had no interaction effect on the kinetics of *in vitro* gas, ruminal fermentation, HCN and *in vitro* degradability. Elemental sulfur supplementation (*P* < 0.05) significantly increased the *in vitro* gas produced from an insoluble fraction (b), *in vitro* DM degradability and either neutral detergent fiber (NDF) or acid detergent fiber (ADF) degradability and propionate (C3) concentration while decreased the ruminal HCN concentration. Urea levels showed a (*P* < 0.05) significant increase of the potential extent of *in vitro* gas production, ruminal ammonia nitrogen (NH_3_-N) and total volatile fatty acid (TVFA). Fresh cassava root supplementation (*P* < 0.05) significantly increased the *in vitro* gas produced from an immediate soluble fraction (a), *in vitro* gas produced from insoluble fraction, *in vitro* gas production rate constant, total VFA, C3 concentration and HCN while decreased ruminal pH, acetate and butyrate concentration. It could be concluded that 2 % elemental sulfur, 4 % urea and 300 mg FCR showed a greater effect on *in vitro* gas production, ruminal fermentation and HCN reduction.

**Conclusions:**

The study found that elemental sulfur, urea, and FCR had no interaction effect on the kinetics of *in vitro* gas, total *in vitro* gas, ruminal fermentation, and HCN concentration. It could be concluded that 2 % elemental sulfur, 4 % urea, and 300 mg FCR showed a greater effect on *in vitro* gas production, ruminal fermentation, and HCN reduction.

## Background

Fresh cassava root (FCR) is one of the main energy source ingredients for ruminant [1] and low price. Global FCR production was 275 million tonnes in 2018 which equals 61.1 % of the total production, and Thailand produced 10.7 % FCR of the total production [2]. The limitation of FCR utilization is due to the presence of hydrogen cyanide (HCN), which is toxic when animals, especially ruminants, consume more than 200 mg/kg fresh matter [3, 4]. Fresh cassava root contains 90 to 114 mg/kg of HCN [3]. The HCN toxicity can be reduced by sun-drying [1]; however, it is not an appropriate method during the rainy season. A chemical method, using sulfur, has been tested and shown to increase thiocyanate concentration, which is less toxic for the host [3, 5, 6]. Briefly, thiocyanate is the product of dependent-sulfur rhodanese enzyme presented in the rumen break-down and subsequently excreted out of the body via urine [7, 8]. Besides its toxicity, FCR has low crude protein (CP) content (2 to 3 %) [9]. Common non-protein nitrogen, urea is added into the diet to increase CP content and use as a nitrogen source for microbial protein synthesis in the rumen [10]. Sulfur is closely related to nitrogen metabolism. Therefore, the optimum level of sulfur supplementation in the diet containing urea is necessary to elucidate. In lambs, improvement of protein utilization efficiency was firstly reported by Johnson et al. [11] when sulfur was added into the diet containing cassava. An *in vitro* study of Promkot et al. [7] similarly reported to significantly increase true protein digestibility, when sulfur of reduced-sodium sulfide nonahydrate was added into a substrate containing cassava foliage and hay. However, a subsequent study by Promkot and Wanapat [8] showed no significant effect of sulfur supplementation on protein digestibility in dairy cows’ diets containing both fresh cassava foliage and cassava hay. In beef cattle, Cherdthong et al. [3] showed no significant effect of feed-block containing sulfur on protein digestibility in a diet composed of the FCR. Supapong and Cherdthong [6] found no significant effect of sulfur in combination with urea on digestibility of dairy cows fed a fermented total mixed ration containing FCR. Insufficient sulfur supply can cause low digestion of dietary nutrients and microbial protein synthesis [12] and its form might significantly affect microbial metabolism in the rumen. National Research Council (NRC) [13] stated that requirement of sulfur for ruminants might be increased for detoxifying HCN. However, the evaluation effect of high sulfur addition into a substrate containing FCR as a source of HCN is still limited and study on the effect of sulfur, urea and FCR and their interaction on *in vitro* gas production, rumen fermentation, and *in vitro* digestibility has not yet been studied.

The study aimed to evaluate the effect of sulfur, urea and FCR at various levels on gas production, ruminal fermentation, and *in vitro* degradability. The study hypothesized that: (1) sulfur, urea and FCR have no interaction effect and (2) effect of FCR and urea is related to sulfur addition.

## Results

### Dietary nutrients

The main energy source of the study diets was dominated by cassava chips. The concentrate contains 12–18 % CP as mainly dominated by urea supplementation at 2 and 4 %. The FCR used in this study contains 104.6 mg/kg of HCN as shown in Table 1.

**Table 1:** Ingredients and chemical composition of concentrate, fresh cassava roots (FCR) and rice straw (% dry matter basis)

Item	0% Sulfur		1% Sulfur		2% Sulfur		FCR	Rice straw
	% Urea	4% Urea	2% Urea	4% Urea	2% Urea	4% Urea		
Ingredients, % dry matter (DM)								
Cassava chip	65	63	64	63	63	61		
Rice bran	10	10	10	10	10	10		
Soybean meal	5	5	5	5	5	5		
Palm kernel meal	15	15	15	14	15	15		
Premix^a^	1	1	1	1	1	1		
Sulfur	0	0	1	1	2	2		
Urea	2	4	2	4	2	4		
Salt	1	1	1	1	1	1		
Molasses	1	1	1	1	1	1		
Chemical composition								
Dry matter, %	93.6	93.6	93.6	93.6	93.6	93.6	33.0	94.7
--------------% DM-----------								
Organic matter	92.8	92.8	92.8	92.8	92.7	92.7	98.5	93.3
Crude protein	12.4	18.1	12.2	18.1	12.5	18.2	2.4	2.7
NDF	12.0	12.1	12.2	12.1	12.1	12.3	53.0	66.7
ADF	8.0	8.2	8.2	8.3	8.3	8.3	31.4	43.5
HCN, mg/kg	-	-	-	-	-	-	104.6	-

^a^ Premix composed of vitamin A: 10,000,000 IU; vitamin E: 70,000 IU; vitamin D: 1,600,000 IU; Fe: 50 g; Zn: 40 g; Mn: 40 g; Co: 0.1 g; Cu: 10 g; Se: 0.1 g; I: 0.5 g. *NDF* means neutral detergent fiber, *ADF* means acid detergent fiber, *HCN* means hydrogen cyanide

### Gas kinetics and total gas production

Table 2 shows the kinetics (a, b, c, and a + b) of *in vitro* gas and cumulative *in vitro* gas at 96 h of incubation. The sulfur, urea and FCR showed no significant interaction effect on the kinetics of gas and total gas production. Sulfur supplementation did not affect total gas and kinetics of gas except gas produced from insoluble fraction (b). Increasing sulfur significantly increased the b value compared to the control; however, 1 and 2 % sulfur supplementation did not differ (Table 2).

**Table 2:** Effect of elemental sulfur (S), urea (U) and fresh cassava root (FCR) on kinetics of gas and gas production at 96 h of incubation

Treatments	S (%)	U (%)	FCR (mg)	Kinetics of gas				Gas production (mL/g)
				a (mL)	b (mL)	c	a+b (mL)	
T1	0	2	0	-4.53	86.05	0.052	81.51	80.92
T2	0	4	0	-5.71	95.65	0.053	89.94	89.61
T3	1	2	0	-3.25	91.53	0.052	98.28	90.41
T4	1	4	0	-5.20	88.84	0.050	83.64	83.91
T5	2	2	0	-3.42	89.60	0.051	86.18	85.52
T6	2	4	0	-6.34	82.63	0.049	76.29	86.50
T7	0	2	200	-8.13	104.20	0.078	96.07	105.95
T8	0	4	200	-7.32	102.11	0.075	94.78	94.38
T9	1	2	200	-9.78	109.43	0.089	99.65	99.64
T10	1	4	200	-8.58	104.78	0.076	96.20	94.80
T11	2	2	200	-9.37	106.90	0.080	97.54	97.51
T12	2	4	200	-9.64	107.32	0.082	97.68	102.62
T13	0	2	300	-10.05	118.63	0.090	108.58	122.35
T14	0	4	300	-11.30	122.37	0.093	111.06	130.75
T15	1	2	300	-11.24	119.00	0.091	107.76	113.20
T16	1	4	300	-11.11	123.97	0.095	112.86	113.86
T17	2	2	300	-11.79	127.79	0.097	116.00	115.68
T18	2	4	300	-12.08	120.93	0.093	108.68	116.62
T19	0	2	400	-11.00	102.46	0.075	91.46	98.45
T20	0	4	400	-11.66	105.56	0.077	93.89	94.87
T21	1	2	400	-11.56	110.58	0.087	99.02	101.96
T22	1	4	400	-12.00	103.93	0.074	91.39	93.73
T23	2	2	400	-11.27	110.64	0.080	99.37	106.31
T24	2	4	400	-12.08	110.60	0.081	98.51	104.51
SEM				1.84	12.20	0.015	9.87	12.40
**S (%)**								
0				-8.71	104.62^b^	0.07	100.91	102.16
1				-9.08	106.44^a^	0.07	99.23	98.93
2				-9.49	107.05^a^	0.07	131.65	101.89
*P*-Value				0.081	0.016	0.110	0.412	0.245
U (%)								
2				-8.78	106.40	0.07	100.64^b^	101.49
4				-9.41	105.67	0.07	142.35^a^	100.50
*P*-Value				0.175	0.282	0.086	<0.0001	0.324
FCR (mg)								
0				-4.74^a^	89.05^c^	0.05^c^	86.14	86.14^c^
200				-8.80^b^	105.79^b^	0.08^b^	99.49	99.15^b^
300				-11.26^c^	122.11^a^	0.09^a^	115.06	118.72^a^
400				-11.59^c^	107.20^b^	0.08^b^	189.19	99.97^b^
*P*-Value				<0.0001	<0.0001	<0.0001	0.367	<0.0001
Interaction								
S*U				0.949	0.103	0.558	0.117	0.217
S*FCR				0.738	0.465	0.764	0.599	0.232
U*FCR				<0.0001	<0.0001	<0.0001	<0.0001	<0.0001
S*U*FCR				0.428	0.776	0.888	0.893	0.076

a means the gas production from the immediately soluble fraction (mL); b means the gas production from the insoluble fraction (mL); c means the gas production rate constant for the degradable fraction *b;* a+b means the potential extent of gas production (mL)

^a,b,c^ means within column showed with different superscript letter accepted significantly different

### Ruminal fermentation, hydrogen cyanide concentration, and protozoal number

The effect of elemental sulfur, FCR and urea on pH, NH_3_-N, HCN and protozoa were shown in Table 3. Elemental sulfur, urea and FCR had no significant interaction effect on pH, NH_3_-N, HCN and protozoal number. The interaction effect between elemental sulfur, FCR and urea has never been elucidated until the present. Elemental sulfur supplementation significantly decreased the HCN concentration but did not affect the pH, NH_3_-N and protozoal number. Sulfur supplementation significantly reduced HCN when compared to the control; however, 1 % vs. 2 % sulfur supplementation did not differ for the HCN reduction.

**Table 3:** Effect of elemental sulfur (S), urea (U) and fresh cassava root (FCR) on rumen pH, ammonia-nitrogen (NH_3_-N), hydrogen cyanide (HCN) and protozoal number

Treatments	S (%)	U (%)	FCR (mg)	pH	NH_3_-N (mg%)	HCN (mg/L)	Protozoa (×10^5^ cell/mL)
T1	0	2	0	6.51	21.1	0.0053	5.5
T2	0	4	0	6.42	21.8	0.0051	5.6
T3	1	2	0	6.48	20.8	0.0050	5.0
T4	1	4	0	6.54	21.9	0.0049	5.4
T5	2	2	0	6.53	20.7	0.0048	4.8
T6	2	4	0	6.55	21.6	0.0048	5.0
T7	0	2	200	6.31	21.3	0.0058	5.2
T8	0	4	200	6.36	22.0	0.0059	5.7
T9	1	2	200	6.28	20.6	0.0055	5.0
T10	1	4	200	6.36	21.6	0.0054	4.9
T11	2	2	200	6.30	20.7	0.0047	5.3
T12	2	4	200	6.32	21.7	0.0046	5.6
T13	0	2	300	6.30	20.6	0.0074	5.2
T14	0	4	300	6.34	22.1	0.0076	5.4
T15	1	2	300	6.28	21.1	0.0057	4.8
T16	1	4	300	6.35	21.5	0.0056	5.3
T17	2	2	300	6.31	21.1	0.0049	5.4
T18	2	4	300	6.34	21.8	0.0048	5.5
T19	0	2	400	6.15	20.1	0.0088	5.5
T20	0	4	400	6.22	21.8	0.0091	5.3
T21	1	2	400	6.18	20.7	0.0076	5.5
T22	1	4	400	6.22	22	0.0079	5.8
T23	2	2	400	6.19	21.1	0.0075	5.4
T24	2	4	400	6.25	21.6	0.0076	5.5
SEM				0.12	0.09	0.001	0.27
S (%)							
0				6.32	21.41	0.006^a^	5.42
1				6.33	21.28	0.005^b^	5.31
2				6.35	21.28	0.005^b^	5.21
*P*-Value				0.771	0.080	<0.0001	0.231
U (%)							
2				6.31	20.84^b^	0.006	5.42
4				6.35	21.81^a^	0.006	5.36
*P*-Value				0.119	<0.0001	0.518	0.189
FCR (mg)							
0				6.50^a^	21.35	0.004^d^	5.21
200				6.32^b^	21.33	0.005^c^	5.29
300				6.31^b^	21.30	0.006^b^	5.26
400				6.20^c^	21.30	0.008^a^	5.50
*P*-Value				<0.0001	0.397	<0.0001	0.406
Interaction							
S*U				0.961	0.002	0.634	0.140
S*FCR				0.632	0.254	<0.0001	0.804
U*FCR				0.036	<0.0001	0.022	0.753
S*U*FCR				0.213	<0.0001	0.746	0.061

^a,b,c,d^ means within column showed with different superscript letter accepted significantly different

### In vitro digestibility

The effect of elemental sulfur, urea and FCR on IVDMD, IVNDFD and IVADFD was shown in Table 4. Elemental sulfur, urea, and FCR had no significant interaction effect on IVDMD, IVNDFD and IVADFD (*P* > 0.05). Elemental sulfur supplementation significantly influenced IVDMD, IVNDFD and IVADFD. The IVDMD, IVNDFD and IVADFD were increased when elemental sulfur was increased. Urea levels did not affect the IVDMD, IVNDFD and IVADFD (Table 4).

**Table 4:** Effect of elemental sulfur (S), urea (U) and fresh cassava root (FCR) on *in vitro* dry matter (IVDMD), *in vitro* neutral detergent fiber degradability (IVNDFD) and *in vitro* acid detergent fiber degradability (IVADFD)

Treatments	S (%)	U (%)	FCR (mg)	IVDMD (%)	IVNDFD (%)	IVADFD (%)
T1	0	2	0	59.56	50.17	27.59
T2	0	4	0	60.80	52.03	28.49
T3	1	2	0	61.64	52.18	27.58
T4	1	4	0	62.73	53.45	28.38
T5	2	2	0	62.65	52.03	28.65
T6	2	4	0	62.96	53.33	29.42
T7	0	2	200	59.69	49.62	28.46
T8	0	4	200	61.95	54.91	28.94
T9	1	2	200	63.98	53.63	28.70
T10	1	4	200	62.77	54.51	29.29
T11	2	2	200	62.60	51.43	30.27
T12	2	4	200	63.58	53.84	31.34
T13	0	2	300	60.82	51.45	29.47
T14	0	4	300	62.18	54.79	30.01
T15	1	2	300	62.48	53.66	29.81
T16	1	4	300	63.01	54.35	30.47
T17	2	2	300	63.44	53.44	31.16
T18	2	4	300	60.80	55.94	31.86
T19	0	2	400	61.30	50.29	28.94
T20	0	4	400	62.69	53.66	29.62
T21	1	2	400	62.21	54.35	29.21
T22	1	4	400	62.79	51.61	29.64
T23	2	2	400	63.07	54.05	30.25
T24	2	4	400	63.07	54.05	30.51
SEM				0.75	1.32	0.27
S (%)						
0				60.82^b^	52.15^b^	29.51^b^
1				62.68^a^	53.78^a^	29.71^b^
2				63.06^a^	53.21^a^	30.51^a^
*P*-Value				<0.0001	0.044	<0.0001
U (%)						
2				61.48^b^	51.95^b^	29.65^b^
4				62.50^a^	54.14^a^	30.17^a^
*P*-Value				0.009	0.0005	0.0001
FCR (mg)						
0				61.72	52.20	29.80
200				62.42	52.99	30.03
300				62.56	54.02	30.05
400				62.06	52.99	29.76
*P*-Value				0.067	0.158	0.159
Interaction						
S*U				0.029	0.150	0.669
S*FCR				0.644	0.959	0.714
U*FCR				0.775	0.845	0.953
S*U*FCR				0.341	0.972	0.956

^a,b,c^ means within column showed with different superscript letter accepted significantly different

### Ruminal volatile fatty acid concentration

The effect of FCR, elemental sulfur, and urea levels on total VFA and their molar portions were shown in Table 5. Interaction between sulfur, urea, and FCR levels was not found for total VFA, C2, C3 and C4 VFA concentrations. Elemental sulfur supplementation significantly affected the C3 concentration but did not affect the total VFA, C2 and C4 concentration. The C3 concentration was increased significantly with the increase of elemental sulfur supplementation, this could be due to the change of VFA products pattern, mainly a decrease of C2 and increase of C3 concentration VFA. Urea levels significantly affected the total VFA but did not influence their molar portions. Fresh cassava root supplementation significantly affected the total VFA and their molar portions (Table 5).

**Table 5:** Effect of elemental sulfur (S), urea (U) and fresh cassava root (FCR) on total volatile fatty acid (VFA) and their molar portions

Treatments	S (%)	U (%)	FCR (mg)	Total VFA	C2	C3	C4	C2:C3
				(mmol/L)	(mol/100 mol)			
T1	0	2	0	74.81	68.68	20.97	10.35	3.28
T2	0	4	0	74.38	68.46	21.26	10.29	3.23
T3	1	2	0	75.21	67.89	22.50	10.42	3.02
T4	1	4	0	74.73	68.12	21.77	10.30	3.13
T5	2	2	0	74.74	67.53	25.43	10.53	2.66
T6	2	4	0	74.35	68.41	25.09	10.50	2.73
T7	0	2	200	76.21	65.44	23.54	10.53	2.78
T8	0	4	200	75.74	66.69	22.66	10.64	2.94
T9	1	2	200	77.41	65.43	26.57	10.50	2.46
T10	1	4	200	77.11	67.11	25.91	10.64	2.59
T11	2	2	200	78.21	66.78	28.45	10.78	2.35
T12	2	4	200	77.76	67.19	27.06	10.75	2.49
T13	0	2	300	77.30	64.00	28.91	10.59	2.21
T14	0	4	300	75.90	64.17	26.83	10.50	2.39
T15	1	2	300	82.72	64.31	29.12	10.57	2.21
T16	1	4	300	80.74	65.11	28.17	10.72	2.31
T17	2	2	300	85.72	64.04	29.89	10.63	2.14
T18	2	4	300	85.24	65.07	29.82	10.60	2.18
T19	0	2	400	74.22	65.48	24.25	10.27	2.77
T20	0	4	400	73.74	66.28	25.52	10.71	2.60
T21	1	2	400	75.22	62.30	26.73	10.98	2.33
T22	1	4	400	74.24	64.37	25.12	10.51	2.56
T23	2	2	400	76.22	64.35	25.11	10.54	2.56
T24	2	4	400	75.74	63.15	25.80	11.05	2.46
SEM				3.37	1.86	2.70	2.22	0.32
S (%)								
0				75.23	66.15	23.92^c^	9.54	2.77
1				77.17	65.57	25.67^b^	8.62	2.56
2				78.49	65.81	27.73^a^	7.16	2.45
*P*-Value				0.421	0.617	<0.0001	0.063	0.081
U (%)								
2				76.59^b^	66.18	25.60	8.40	2.63
4				77.33^a^	65.52	25.96	8.49	2.55
*P*-Value				0.008	0.174	0.337	0.872	0.146
FCR (mg)								
0				74.70^c^	68.18^a^	22.75^c^	8.98^ab^	2.98^a^
200				77.08^b^	66.43^b^	26.23^b^	7.78^bc^	2.60^b^
300				81.19^a^	64.45^c^	28.54^a^	6.75^c^	2.24^c^
400				74.89^c^	64.32^c^	25.56^b^	10.26^a^	2.54^b^
*P*-Value				<0.0001	<0.0001	<0.0001	0.0004	<0.0001
Interaction								
S*U				0.717	0.713	0.884	0.996	0.436
S*FCR				<0.0001	0.449	0.017	0.018	0.249
U*FCR				0.437	0.939	0.980	0.904	0.772
S*U*FCR				0.958	0.908	0.864	0.791	0.861

C2 means acetic acid; C3 means propionic acid; C4 means butyric acid; C2:C3 means acetic acid to propionic acid ratio

^a,b,c^ means within column showed with different superscript letter accepted significantly different

## Discussion

### *In vitro* gas kinetics and total gas production

The b value represents the gas produced from the insoluble fraction. Therefore, the increase of the b value suggested that sulfur supplementation could improve the digestion of fiber. Morrison et al. [14] stated that sulfur supplementation could improve the microbial activity in the rumen, mainly anaerobic fungi by stimulating the excretion of the fibrous breakdown enzyme. A similar result was reported by Promkot et al. [7] who, significantly found an increase of the b value when increased sulfur supplementation up to 1 % in substrate containing cassava (foliage and hay). Urea levels in concentrate significantly increased the potential extent of *in vitro* gas production (a + b), in which 4 % urea showed significantly higher than 2 % urea. A similar finding was reported by Lunsin et al. [15] who found 5 % urea increased the a + b value compared to 0 % urea. However, the mechanism of this improvement is not clear. Hameed et al. [16] assumed that the greater kinetics of gas could be contributed by the greater structural carbohydrate degradation with urea treatment, which could clearly see a greater *in vitro* NDF and ADF degradability when increased urea levels (Table 4). Fresh cassava root supplementation significantly affected the kinetics of gas except the a + b value and total gas (Table 2). Increasing FCR supplementation significantly increased *in vitro* gas produced from immediate soluble fraction (a), b value, *in vitro* gas production rate constant for insoluble fraction (c), and total gas; whereas the highest kinetics of gas and total gas was found with 300 mg of FCR supplementation. This could be explained by the more available carbohydrate as FCR increased came to the rumen for microbial fermentation resulting in greater kinetics of gas and total gas. Promkot et al. [7] used cassava foliage and hay in the substrate did not affect the kinetics of gas and total gas, this might be due to the low soluble carbohydrate content in cassava foliage and hay compared to the FCR. Dagaew et al. [17] reported that reduced FCR levels in the substrate significantly decreased the kinetics of gas and total gas.

### Ruminal fermentation, hydrogen cyanide concentration, and protozoal number

The reduction of the HCN could be explained by the action of rhodanese enzyme presented in the rumen that converts HCN into a less toxic substance (thiocyanate) and excreted out via urine [6, 7]. Promkot et al. [7] found that an increase of sulfur supplementation at 0.5 and 1 % into the fresh cassava foliage substrate showed a great *in vitro* disappearance of HCN compared to 0.2 % of sulfur supplementation. Similarly, Dagaew et al. [17] added sulfur into feed-block at 2 and 4 % with FCR supplementation showed a significant decrease of the *in vitro* HCN concentration. Promkot and Wanapat [8] found an increase of milk thiocyanate in dairy cows fed fresh cassava foliage and hay when increased sulfur supplementation from 0.15 to 0.4 %. Supapong and Cherdthong [6] found a significant increase in milk thiocyanate concentration in dairy cows fed a total mixed ration containing FCR when increased sulfur supplementation from 1 to 2 %. Urea levels significantly influenced the NH_3_-N concentration but did not affect pH, HCN concentration, and protozoal number. Increasing urea significantly increased the concentration of NH_3_-N, this could be due to the activity of urease enzyme produced by the ruminal microbes to degrade urea into ammonia which, subsequently used for microbial protein synthesis [10]. Supapong and Cherdthong [6] found a significantly higher NH_3_-N concentration with 2.5 % than 1.25 % urea in dairy cows fed total mixed ration. Wanapat et al. [18] fed dairy cows with 5.5 % urea-treated rice straw resulting in the highest NH_3_-N concentration when compared to the control and 2.2 % urea treatment. FCR supplementation significantly affected the ruminal pH and HCN concentration but did not affect NH_3_-N and protozoal numbers (Table 3). An increase in FCR supplementation significantly decreased the ruminal pH while increased the HCN concentration. A decrease of ruminal pH when increased FCR supplementation could be due to the accumulation of lactic acid from carbohydrate fermentation by ruminal microbes. The greater lactate accumulation led to a lower pH in the rumen. As FCR contained HCN, therefore increase of FCR supplementation in the substrate resulted in the greater HCN concentration in the ruminal fluid. Dagaew et al. [17] varied FCR ratio with rice straw did not affect the ruminal pH but significantly increased the ruminal HCN concentration. Cherdthong et al. [3] fed FCR at 1 and 1.5 % body weight did not change the ruminal pH of Thai native beef cattle but significantly increased the blood thiocyanate concentration after 4 h post-feeding. Promkot and Wanapat [8] fed dairy cows with cassava foliage and hay did not alter the ruminal pH but significantly increased the serum and milk thiocyanate.

### *In vitro* digestibility

The interaction effect of elemental sulfur, urea, and FCR has never been evaluated until the present. However, the interaction effect of elemental sulfur and FCR have been evaluated and found no interaction effect on both *in vitro* and *in vivo* studies [3, 17]. Supapong and Cherdthong [6] evaluated the interaction effect of elemental sulfur and urea and found no interaction effect on digestibility. Elemental sulfur supplementation significantly influenced IVDMD, IVNDFD and IVADFD. The IVDMD, IVNDFD and IVADFD were increased when elemental sulfur was increased. The increase of IVDMD, IVNDFD and IVADFD might be due to the benefits of sulfur in enhancing the ruminal microbial activity on digestion. Slyter et al. [19] stated that sulfur could increase cellulolytic bacteria, and may improve fiber degradability. Dagaew et al. [17] found a significant increase of IVDMD with feed-block containing elemental sulfur but did not found a significant effect on IVNDFD and IVADFD. Similarly, Cherdthong et al. [3] found significant increased apparent DM digestibility in Thai native beef cattle fed feed-block containing sulfur but did not found for apparent fiber digestibility. Promkot et al. [7] revealed an increase of *in vitro* true digestibility with sulfur supplementation in substrate containing both cassava foliage and hay. A later study by Promkot and Wanapat [8] in dairy cows found that sulfur supplementation significantly affected only DM digestibility but did not affect the fiber digestibility. Urea levels did not affect the IVDMD, IVNDFD, and IVADFD (Table 4). A similar finding was reported by Boucher et al. [20] who found no change of nutrient digestibility with urea supplementation into corn silage diet for dairy cows. The lack of urea effect on *in vitro* degradability in this study could be related to the maximum ruminal NH_3_-N concentration to support the maximal ruminal digestibility. The NH_3_-N concentration in this study ranged from 20 to 21 mg/dl (Table 3). Boucher et al. [20] found that 9 mg/dl of ruminal NH_3_-N would be more than adequate for supporting the maximal ruminal DM digestibility. Kang-Meznarich and Broderick [21] revealed that 3.3 mg/dl was adequate for the maximal DM digestibility in non-lactating dairy cows fed pelleted diet. Chanjula and Ngampongsai [22] found that increase in urea supplementation (0 to 3 %) in concentration did not affect the apparent nutrient digestibility in growing goats fed elephant grass. FCR supplementation did not affect the IVDMD, IVNDFD, and IVADFD (Table 4). Promkot et al. [7] found that used cassava foliage and hay in the substrate did not affect the *in vitro* true digestibility. A later study by Promkot and Wanapat [8] similarly found no effect of cassava foliage and hay on apparent nutrient digestibility in dairy cows. Cherdthong et al. [3] found that 1 and 2 % cassava root supplementation did not affect the apparent nutrient digestibility in Thai native beef cattle.

### Ruminal volatile fatty acid concentration

The interaction effect of elemental sulfur, urea, and FCR was the lack in the literature until the present. However, the interaction effect of elemental sulfur and urea has been evaluated and found no interaction effect on total VFA and their molar portions [6]. And the interaction effect of FCR and sulfur has been reported by Dagaew et al. [17] and Cherdthong et al. [3] who found no interaction effect between sulfur and FCR on total VFA and their molar concentration. Elemental sulfur supplementation significantly affected the C3 concentration but did not affect the total VFA, C2, and C4 concentration. The C3 concentration was increased significantly with the increase of elemental sulfur supplementation, this could be due to the change of VFA products pattern, mainly a decrease of C2 and increase of C3 concentration. Thompson et al. [23] revealed that dietary containing sulfur decreased the C2 to C3 ratio resulting in a greater C3 concentration. Dagaew et al. [17] found an increase of *in vitro* C3 concentration when increased sulfur levels in the feed-block. Supapong and Cherdthong [6] found an increase of ruminal C3 concentration with sulfur supplementation at 1 and 2 % in dairy cows fed a total mixed ration containing FCR. Promkot et al. [7] found a trend in increasing ruminal C3 concentration in dairy cows fed cassava foliage and hay in the diet. Urea levels significantly affected the total VFA but did not influence their molar portions (Table 5). An increase of urea showed an increase in the total VFA. This may be due to the effect of urea on carbohydrate metabolism in the rumen. Obara [24] revealed that used urea as a nitrogen source could enhance the ruminal microbes’ activity to digest carbohydrates resulting the greater VFA production. Similar findings for an increase of total VFA with urea treatment have been reported [6]. Fresh cassava root supplementation significantly affected the total VFA and their molar portions (Table 5). The total VFA and C3 concentration were increased when increased the FCR supplementation; in contrast, C2 and C4 were decreased when increased the FCR supplementation. The higher total VFA and C3 concentration and lower C2 and C4 concentration were found in substrate containing FCR compared to the control. Increasing C3 concentration normally decreases the C2 and C4 concentration in the rumen because most carbohydrate fermentation by microbes in the rumen resulting in the greater C3 concentration. Notably, the increase of FCR up to 400 mg significantly decreased the total VFA and C3 concentration while significantly increased C4 concentration when compared with the 300 mg of FCR supplementation. This might be due to the negative effect of HCN on ruminal microbes’ activity when supplemented up to 400 mg of the total substrate. Cherdthong et al. [3] found an increase of the C3 concentration in Thai native beef cattle when increased cassava root from 1 to 2 % of body weight. Similarly, Dagaew et al. [17] found an increase of the *in vitro* C3 concentration when increased FCR ratio with rice straw in the substrate.

## Conclusions

The study found that elemental sulfur, urea and FCR had no interaction effect on the kinetics of gas, total gas, ruminal fermentation, and HCN concentration. Elemental sulfur supplementation significantly increased the gas produced from insoluble fraction, *in vitro* degradability, and C3 concentration while decreased the ruminal HCN concentration. Urea levels showed a significant increase in the potential extent of gas production, ruminal NH_3_-N and total VFA. FCR supplementation significantly increased the kinetics of gas except for the potential extent of gas and *in vitro* total gas, total VFA, C3 concentration and HCN while decreased ruminal pH, C2 and C4 concentration. It could be concluded that 2 % elemental sulfur, 4 % urea, and 300 mg FCR showed a greater effect on gas production, ruminal fermentation and HCN reduction. However, *in vivo* study is needed to be conducted to elucidate their further effect. Sulfur levels (1 % vs. 2 %), urea levels (2 % vs. 4 %) and FCR levels (300 mg vs. 400 mg) will be selected to study in the *in vivo* studies.

## Methods

Animal ethics approval (ACUC-KKU 32/61) was issued to ensure standard care of animals during the study.

### Experimental design and treatments

A 3 × 2 × 4 in a completely randomized design were conducted. Factor A was level of sulfur at 0 %, 1 and 2 % of concentrate dry matter (DM), factor B was level of urea at 2 and 4 % of concentrate DM, and factor C was level of the FCR at 0, 200, 300 and 400 mg DM of the total substrate. The FCR (*Manihot esculenta* Kasetsart 50) at one-year-old of age was purchased from a local supplier located in Khon Kaen province, Thailand. Sulfur and urea were purchased commercially. Sulfur was in powder form and obtained from S.P. Science Company located in Khon Kaen province, Thailand.

### Substrate preparation

The substrates including rice straw and concentrate mixture were dried at 60 °C and ground to pass a 1-mm sieve (Cyclotech Mill, Tecator, Sweden), while FCR was used as a fresh form. The ground samples of FCR, rice straw, and concentrate mixture were used to analyze DM (ID 967.03), organic matter (OM, ID 942.05) and crude protein (CP, ID 984.13) using the method of AOAC [25], neutral detergent fiber (NDF) and acid detergent fiber (ADF) according to Van Soest et al. [26]. Content of HCN in FCR was analyzed by using spectrophotometry (SpectroSC, LaboMed, inc, USA) with the 2,4-quinolinediol-pyridine reagent [27]. The concentrate ingredients and chemical compositions of concentrate, rice straw and FCR used in this study were provided in Table 1.

### Animals and rumen fluid provision

Two male rumen-fistulated dairy steers with body weight (BW) of 400 ± 50 kg were raised in a separate pen with accessible clean water and fed concentrate at 0.5 % BW/day. The concentrate was formulated to have 12 % CP following the recommendation of NRC [28]. Rice straw was daily fed *ad libitum*. The feeding lasted for 14-days before ruminal fluid was collected. After 14-days of feeding, approximately 1500 mL of ruminal fluid were manually collected and filtered through cheesecloth (four-layers) into pre-warmed thermos flasks, then immediately transferred to the laboratory.

### Inoculum preparation and in vitro fermentation

The inoculum was made of the ruminal fluid and artificial saliva. The artificial saliva was prepared according to Menke and Steingass [29]. A 1:2 ratio of ruminal fluid and artificial saliva was mixed in a thermos flask to form the inoculum, warmed at 39 °C, and continuously supplied with carbon dioxide. A 369 serum bottles (150 mL volume) were prepared, in which 72 serum bottles with 3 bottles for blank were used to study the kinetics of gas, 147 bottles used to study ruminal fermentation (pH, ammonia nitrogen-NH_3_-N, VFA and protozoa) at 4 and 6 h of incubation, and 147 bottles used to study the degradability at 12 and 24 h of incubation. All treatments were done in three replications. The ground concentrate mixture and rice straw were weighed into the serum bottles at 50:50 ratio to obtain the final substrate of 500 mg. The ground FCR (fresh form) was weighed into the bottles at its respective levels of total substrate. A 50 mL of artificial inoculum was withdrawn and injected into the serum bottles containing their respective treatments’ substrate. The bottles were then transferred to the water bath with pre-set temperature of 39 °C and incubated at various time series.

### Sample collection and analysis

The *in vitro* gas produced from fermentation was manually measured using a pressure transducer syringe at 0, 0.5, 1, 2, 4, 6, 8, 12, 18, 24, 48, 72 and 96 h of incubation. The amount of gas at each time of incubation was fitted to the *in vitro* gas equation of Ørskov and McDonald [30] to study the kinetics of gas as follows:

$$ y=a+b\left[1-{e}^{\left(- ct\right)}\right] $$

where a is the *in vitro* gas production from the immediately soluble fraction, b is the *in vitro* gas production from the insoluble fraction, c the *in vitro* gas production rate constant for the insoluble fraction (b), a + b is the potential extent of *in vitro* gas production, and t the incubation time.

After incubated for 4 and 6 h, the pH was measured using a Hanna pH meter (model HI83141, HANA instruments, Romania) from 147 bottles, and the liquid samples were then filtered through cheesecloth (four-layers) and centrifuged at 16.000× *g* for 15 min. After centrifuged, the supernatant was collected by dividing into two parts: the first part was used to analyzed NH_3_-N concentration using Kjeldahl methods according to AOAC [25] and VFA proportions including acetate (C2), propionate (C3), and butyrate (C4) using high-performance liquid chromatography (Instruments by controller water model 600E, Water model 484 UV detector, column Novapak C18, column size 4 × 150 mm, mobile phase 10 mM H_2_PO_4_ (pH 2.5); ETL Testing Laboratory, Inc., Cortland, NY). The remaining part was mixed with formaldehyde at 1:9 ratio for protozoal counts using microscopic (Boeco, Hamburg, Germany). Hydrogen cyanide concentration in the liquid samples was measured by using spectrophotometry [27].

After incubated for 12 and 24 h, the samples were collected by filtering through pre-weighed Gooch crucibles, then the Gooch crucibles containing sample were oven-dried at 60 °C for 24 h. After oven-dried, the DM of samples and blank was used to calculate the *in vitro* DM degradability (IVDMD) [31]. Then, the samples were analyzed for *in vitro* NDF and ADF degradability according to Van Soest et al. [26].

### Statistical analysis

All data were subjected to the General Linear Models (GLM) procedures of SAS [32]. The following model was used:

$$ {\mathrm{y}}_{\mathrm{i}\mathrm{j}\mathrm{k}\mathrm{l}}=\upmu +{\mathrm{a}}_{\mathrm{i}}+{\mathrm{b}}_{\mathrm{j}}+{\mathrm{c}}_{\mathrm{k}}+{\mathrm{a}\mathrm{b}}_{\mathrm{i}\mathrm{j}}+{\mathrm{a}\mathrm{c}}_{\mathrm{i}\mathrm{k}}+{\mathrm{b}\mathrm{c}}_{\mathrm{j}\mathrm{k}}+{\mathrm{a}\mathrm{b}\mathrm{c}}_{\mathrm{i}\mathrm{j}\mathrm{k}}+{\upvarepsilon}_{\mathrm{i}\mathrm{j}\mathrm{k}\mathrm{l}} $$

where y is the observation, m is the overall mean, a_i_ is the level of sulfur(i,1–3), b_j_ is the level of urea (j, 1–2), c_k_ is the level of FCR at 0 %, 40 %, 60 and 80 % of all diet (k,1–4), ab_ij_, ac_ik_, bc_jk_, abc_ijk_, is the interaction effect and ɛ_ijkl_ is the error. Differences among treatment means for all parameters were contrasted by Tukey’s Multiple Comparison Test. Differences among means were accepted at *P* < 0.05.

## Data Availability

The dataset generated and/or analyzed during the current study is not publicly available since the data is a preliminary part of another study. The data is, however, available from the corresponding author on reasonable request.

## Abbreviations

°C: Degree celsius
ADF: Acid detergent fiber
AOAC: The Association of Official Analytical Chemists
C2: Acetate
C3: Propionate
C4: Butyrate
CH4: Methane
CP: Crude protein
DM: Dry matter
FCR: Fresh cassava root
HCN: Hydrogen cyanide
IVADFD: *In vitro* acid detergent fiber degradability
IVNDFD: *In vitro* neutral detergent fiber degradability
IVDMD: *In vitro* dry matter
N: Nitrogen
NDF: Neutral detergent fiber
OM: Organic matter
RS: Rice straw
S: Sulfur
U: Urea
VFA: Volatile fatty acids
